# In Vivo and In Vitro Activities and ADME-Tox Profile of a Quinolizidine-Modified 4-Aminoquinoline: A Potent Anti-*P. falciparum* and Anti-*P. vivax* Blood-Stage Antimalarial

**DOI:** 10.3390/molecules22122102

**Published:** 2017-12-01

**Authors:** Nicoletta Basilico, Silvia Parapini, Anna Sparatore, Sergio Romeo, Paola Misiano, Livia Vivas, Vanessa Yardley, Simon L. Croft, Annette Habluetzel, Leonardo Lucantoni, Laurent Renia, Bruce Russell, Rossarin Suwanarusk, Francois Nosten, Giulio Dondio, Chiara Bigogno, Daniela Jabes, Donatella Taramelli

**Affiliations:** 1 Department of Biomedical, Surgical and Dental Sciences (DiSBIOC), University of Milan, Via Pascal 36, 20133 Milan, Italy; nicoletta.basilico@unimi.it; 2 Department of Pharmacological & Biomolecular Sciences (DiSFeB), University of Milan, Via Pascal 36, 20133 Milan, Italy; silvia.parapini@unimi.it (S.P.); paola.misiano@unimi.it (P.M.); 3 Department of Pharmaceuticals Sciences (DISFARM), University of Milan, Via Mangiagalli 25, 20133 Milan, Italy; anna.sparatore@unimi.it (A.S.); sergio.romeo@unimi.it (S.R.); 4 Department of Immunology and Infection, Faculty of Infectious and Tropical Diseases, London School of Hygiene and Tropical Medicine (LSHTM), Keppel Street, London WC1E 7HT, UK; livia.vivas.net@gmail.com (L.V.); Vanessa.Yardley@lshtm.ac.uk (V.Y.); simon.croft@lshtm.ac.uk (S.L.C.); 5 School of Pharmacy, University of Camerino, Via d’Accorso 16, 63032 Camerino, MC, Italy; annette.habluetzel@unicam.it (A.H.); l.lucantoni@griffith.edu.au (L.L.); 6 Singapore Immunology Network (SIgN), Agency for Science Technology and Research, Biopolis, Singapore 138648, Singapore; renia_laurent@immunol.a-star.edu.sg (L.R.); b.russell@otago.ac.nz (B.R.); noi.suwanarusk@otago.ac.nz (R.S.); 7 Shoklo Malaria Research Unit, Mahidol-Oxford Tropical Medicine Research Unit, Faculty of Tropical Medicine, Mahidol University, Mae Sot 63110, Thailand; francois@tropmedres.ac; 8 Centre for Tropical Medicine and Global Health, Nuffield Department of Medicine Research building, University of Oxford Old Road Campus, Oxford OX3 7FZ, UK; 9 Aphad Srl, Via della Resistenza 65, 20090 Buccinasco, Milan, Italy; G.DONDIO@aphad.eu (G.D.); c.bigogno@aphad.eu (C.B.); 10 NeED Pharmaceuticals Srl, Viale Ortles 22/4, 20139 Milan, Italy; jabesdaniela@gmail.com

**Keywords:** malaria, 4-aminoquinoline, drug resistance, *P. falciparum*, *P. vivax*

## Abstract

Natural products are a prolific source for the identification of new biologically active compounds. In the present work, we studied the in vitro and in vivo antimalarial efficacy and ADME-Tox profile of a molecular hybrid (AM1) between 4-aminoquinoline and a quinolizidine moiety derived from lupinine (*Lupinus luteus*). The aim was to find a compound endowed with the target product profile-1 (TCP-1: molecules that clear asexual blood-stage parasitaemia), proposed by the Medicine for Malaria Venture to accomplish the goal of malaria elimination/eradication. AM1 displayed a very attractive profile in terms of both in vitro and in vivo activity. By using standard in vitro antimalarial assays, AM1 showed low nanomolar inhibitory activity against chloroquine-sensitive and resistant *P. falciparum* strains (range IC_50_ 16–53 nM), matched with a high potency against *P. vivax* field isolates (Mean IC_50_ 29 nM). Low toxicity and additivity with artemisinin derivatives were also demonstrated in vitro. High in vivo oral efficacy was observed in both *P.* *berghei* and *P*. *yoelii* mouse models with IC_50_ values comparable or better than those of chloroquine. The metabolic stability in different species and the pharmacokinetic profile in the mouse model makes AM1 a compound worth further investigation as a potential novel schizonticidal agent.

## 1. Introduction

Malaria is an infectious disease caused by *Plasmodium* parasites; two species, *P. falciparum* and *P. vivax*, are responsible for more than 400,000 deaths/year worldwide. The majority of fatalities (90%), mostly children below the age of five and pregnant women, occur in Africa, and in particular in the sub-Saharan regions [1].

Increased funding and efforts to find new, effective treatments, together with improvements in the use of control tools (insecticide-treated nets and residual indoor spraying), succeeded in reducing malaria incidence by 41% between 2000 and 2015. Similarly, global malaria mortality decreased by 62% over the same years. However, the emergence of parasite resistance to the current combination therapies based on artemisinin derivatives (ACTs) may frustrate all these efforts [2,3,4,5]. Moreover, in some of the countries where *P. falciparum* has been eliminated (Central Asia, Argentina, Belize, Mexico, and large parts of China) there is growing evidence for an increase in morbidity and mortality due to *P. vivax* infection [6,7,8]. For these reasons, the discovery and development of new drugs active against different *Plasmodium* species remains a high priority. 

The 4-aminoquinoline class of drugs, represented by the classical antimalarial agent chloroquine (CQ), has been highly successful in the treatment of malaria for decades before resistance emerged and spread. Another member of the family, amodiaquine (AQ), is still used in ACT [9]. For their stability, bioavailability, and efficacy, quinoline-type drugs are extremely effective as blood-stage schizonticidal agents. However, their usage is hampered by the large diffusion of resistance of *P. falciparum* and *P. vivax* strains to CQ, and cross-resistance to AQ and mefloquine. In countries where CQ use was discontinued, studies have shown that, in the absence of drug pressure, CQ-resistant strains of *P. falciparum* have been replaced by CQ-sensitive isolates [10]. Therefore, the development of CQ analogues as multi-species antimalarial agents may still be valid and has been recently highlighted and reviewed [11,12].

Chemical modification of the side chain of 4-aminoquinolines, either in length or with the introduction of bulky basic groups, has been shown to overcome CQ resistance. Over the years, several compounds active also in CQ-resistant strains have been synthesized and some of them, including one from our group, reached pre-clinical testing [13,14,15]. Ferroquine and AQ 13, for example, are in advanced clinical phase II evaluation [16,17,18,19,20].

In order to eliminate malaria, the antimalarial target product profiles have been recently revised by the Medicine for Malaria Venture (MMV), highlighting the importance of developing compounds that will be able to target different parasite species and stages, maintaining a good ADME/PK profile which can be relevant for combination therapies [21].

**Figure 1:**
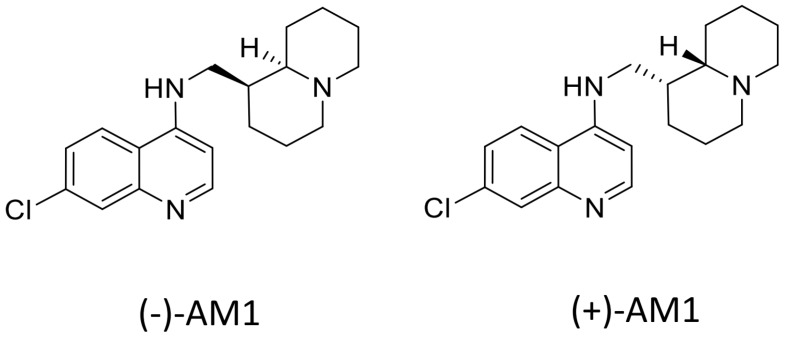
Structure of investigated compounds: 7-chloro-*N*-(((1*S*,9a*R*)-octahydro-1*H*-quinolizin-1-yl)methyl)quinolin-4-amine (−)-AM1; 7-chloro-*N*-(((1*R*,9a*S*)-octahydro-1*H*-quinolizin-1-yl)methyl)quinolin-4-amine (+)-AM1.

A few years ago, we synthesized and studied a new series of 7-chloro-4-aminoquinolines characterized by the presence of a bulky quinolizidinyl or quinolizidinylalkyl side chain, the latter derived from the natural product (−)-lupinine, an alkaloid extracted from *Lupinus luteus* seeds [22]. Among them, 7-chloro-4-(*N*-lupinyl)aminoquinoline, named (−)-AM1, was selected for further physicochemical and biological characterization. The results indicated that the antiplasmodial activity of (−)-AM1 was likely due to inhibition of hemozoin formation. This was directly tested in a beta-hematin inhibitory assay in vitro, and it was substantiated by measuring the lipophilicity of (−)-AM1 [23]. In fact, (−)-AM1 is more lipophilic than chloroquine and thus able to accumulate at the interface between lipids and water where hemozoin formation is reported to occur [24]. The efficacy against CQ-resistant strains of *P. falciparum* could be ascribed to the ability of (−)-AM1 to block drug efflux by hydrophobic interactions with *Pf*CRT, the transporter responsible for CQ resistance [23]. Due to the limited market supply of (−)-lupinine, the total synthesis of 7-chloro-4-(*N*-lupinyl)aminoquinoline, the most active compound of the series, was developed resulting in a racemic product called (±)-AM1 (Figure 1) [25].

Both the racemate and its purified enantiomers showed potent antimalarial in vitro activity against both CQ-S (D10) and CQ-R (W-2) strains of *P. falciparum*, and a good therapeutic index [25].

In the present work, we extended the previous observations by completing in vivo efficacy studies of racemic AM1 and its purified enantiomers in two rodent malaria models (*P. berghei* and *P. yoelii*) with different susceptibilities to CQ, and activity studies against *P. vivax* clinical isolates. We also evaluated the ADME-Tox profiles in vivo and in vitro.

## 2. Results

### 2.1. In Vitro Antimalarial Activity against P. falciparum and P. vivax

Racemic AM1 and its purified enantiomers (Figure 1) showed an antimalarial activity in vitro in the low nanomolar range (IC_50_ = 16–53 nM) when tested against both CQ-S (D10) and CQ-R strains of *P. falciparum* (FCR1 and FCR3), including a multidrug-resistant parasite TM91C235 (isolated in Thailand) (Table 1). All the compounds had very low toxicity and an excellent selectivity index (SI > 1000) against human endothelial cells and fibroblasts, and mouse cell lines, as previously reported [25]. Furthermore, (±)-AM1 showed remarkable antimalarial activity against 10 different field isolates of *P. vivax* with potency about 3 times higher than that of CQ (Table 1, right column).

**Figure 2:**
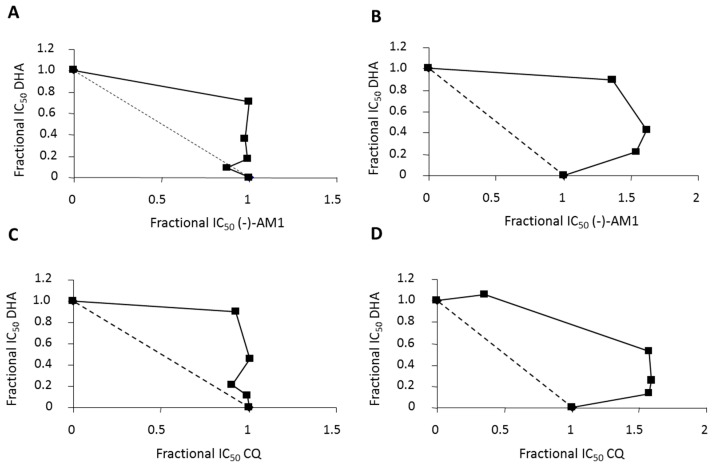
Isobologram analysis of the antimalarial activity of (−)-AM1 in combination with dihydroartemisinin (DHA) against D10 chloroquine-sensitive (CQ-S) (**A**) or W2 chloroquine-resistant (CQ-R) (**B**) *P. falciparum* strains. Combinations of DHA with chloroquine (CQ) are shown in (**C**,**D**). DHA fractional IC_50_ values were calculated by dividing the IC_50_ of DHA combined with (−)-AM1 or CQ, respectively, by the IC_50_ of DHA alone, and plotted on the horizontal axis. The corresponding fractional (−)-AM1 or CQ IC_50_ values were calculated by dividing each fixed concentration by the IC_50_ of the (−)-AM1 or CQ alone, and plotted on the vertical axis. An isobologram close to the diagonal indicates an additive effect. Curves significantly (>2) above or below the diagonal indicate antagonistic or synergistic effects, respectively.

When tested in combination studies with dihydroartemisinin (DHA) using D10 (CQ-S) and W-2 (CQ-R) parasites, the isobolograms showed that (−)-AM1 had additive activity with DHA (FIC < 2), with a non-significant trend toward antagonism (Figure 2A,B). The results are similar to those obtained with CQ (Figure 2C,D).

### 2.2. In Vivo Oral Efficacy Studies

Racemic AM1, (−)-AM1, and (+)-AM1 were tested for oral in vivo efficacy in the four-day mouse infection model against *P. berghei* ANKA [26] and *P. yoelii*. The data, expressed as ED_50_ and ED_90_ values, are reported in Table 2.

All compounds showed very good oral efficacy, similar to that of CQ. The ED_50_ did not differ significantly between the two enantiomers, whereas the ED_90_ of (+)-AM1 was lower than that of (−)-AM1 and the racemate, and identical to that of CQ. In the *P. yoelii* model, which is considered relatively CQ-resistant, the (−)-AM1 enantiomer maintained its antimalarial activity, while CQ had lower efficacy, as expected. The maximum tolerated dose, MTD, in CD1 mice treated with (−)-AM1 was greater than 100 mg/kg, i.p. No toxic clinical signs or mortality were noted up to this dose. Similar results were obtained using the compounds as free bases or as water soluble salts, confirming a therapeutic index greater than that observed for CQ (MTD 50 mg/kg i.p.).

### 2.3. In Vivo Transmission-Blocking and Prophylactic Effects

Having demonstrated a high in vivo oral efficacy against asexual blood-stages in the four-day test, we investigated the activity of the (−)-AM1 enantiomer on the transmission stages of the rodent malaria parasite *P. berghei* ANKA and *Anopheles stephensi* mosquitoes. BALB/c mice, four days after infection with *P. berghei*, were treated orally with 50 mg/kg of (−)-AM1 and anesthetized and submitted to the bites of *A. stephensi* mosquitoes one hour later. After 10 days, a high proportion (86.4%) of mosquitoes infected with gametocytes from (−)-AM1-treated mice hosted oocysts on their midgut (CI_95_ 81.4–103.6%). This was not different from control mosquito positivity (90%, CI_95_ 80.6–99.4%). Similarly, there was not a significant difference in infection intensity, i.e., the number of oocysts per midgut, between mosquitoes infected with gametocytes from (−)-AM1-treated mice or controls: an average of 54.11 (CI_95_ 39.77–68.45) and 39.17 (CI_95_ 18.44–59.89) oocysts were found, respectively (Student’s *t*-test *p* = 0.24). 

When tested for prophylactic activity (see M&M for details), no significant differences in parasitaemia in (−)-AM1 treated mice compared to control mice were seen during the entire evaluation period (days 5–9 post-infection). It can be concluded that in the present model, (−)-AM1 does not show transmission-blocking or prophylactic activity.

### 2.4. Metabolic Stability and Biotransformation Studies in Rat, Mouse, and Human Microsomes

**Figure 3:**
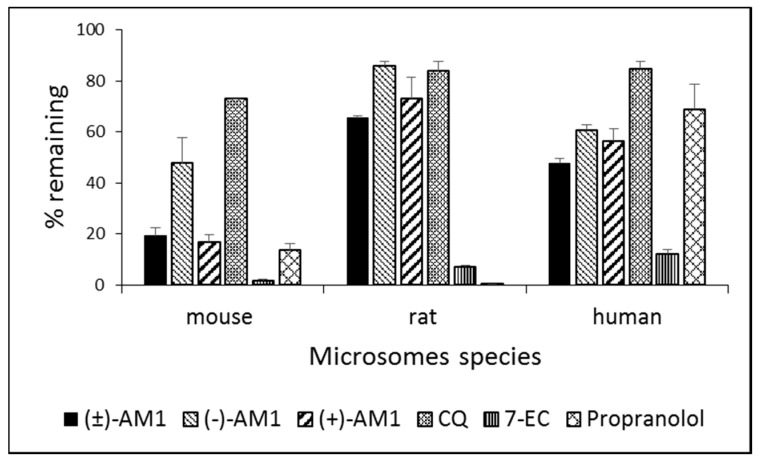
Metabolic stability studies in mouse, rat, and human microsomes. Hepatic metabolic stability of the AM1 racemate and its enantiomers was determined in the microsomes of mouse, rat, and human species after 30 min incubation. 7-EC and propranolol were used as reference compounds. Results are expressed as Mean ± S.D., *n* = 2. Metabolic stability range values: >50: High; 10–50: Medium; <10: Low.

Preliminary experiments were then conducted to explore the ADME properties of these compounds. When evaluated in hepatic microsomes (Figure 3), racemic AM1 and its enantiomers showed high metabolic stability (greater than 50% remaining at 30 min) in both rat and human species, comparable to that of CQ. On the contrary, in the mouse microsomes, all the compounds were moderately unstable (from 16 to 47% remaining at 30 min), with racemic AM1 and (+)-AM1 less stable than (−)-AM1. Standard reference compounds propranolol and 7-ethoxycoumarin were metabolized as expected [27].

Biotransformation studies were performed only on (−)-AM1 in rat, mouse, and human hepatic microsomes in order to identify major metabolites and highlight possible differences among species.

A representative chromatogram in the mouse and the MS spectra are shown in Figure S1 (Supplementary Materials), while the proposed structures of metabolites in all the species, together with the retention times, are shown in Table 3. In the same table, the metabolites found in mouse plasma after 50 mg/kg oral treatment are included. The retention times and [M + H]^+^ of the metabolites are the same for all three species.

The metabolites of (−)-AM1 ([M + H]^+^ = 330 at 6.1 min) were detected in all species but with different abundance, in agreement with the microsomal stability data. A mass [M + H]^+^ = 346 is observed at 4.7 min and 5.5 min (not present in humans, data not shown) suggesting the presence of a hydroxylated or a protonated *N*-oxide metabolite, further confirmed by the fragment *m*/*z* = 329 (M-17). All the fragmentations detected are referred to the octahydroquinolizine portion of the parent compound (i.e., *m*/*z* 138 and 152, see Figure S2). Fragment *m*/*z* 182 is related to the hydroxylated octahydroquinolizine portion, which includes the methylenamino group. The fragment *m*/*z* 194 seems related to the chloroquinoline including the methylenamino bridge (Figure S3). Together, these data may suggest that the possible metabolite could be the *N*-oxide on the 4-amino-7-chloroquinoline. Fragment *m*/*z* 202.7, whose structure is not elucidated, can probably be referred to the chloroquinoline portion (see Figure S3). The concentration of the metabolite at 5.5 min (hydroxy or *N*-oxide) identified in rat and mouse microsome profiles was too small to be identified by a MS/MS analysis.

The MS/MS spectra of a minor metabolite [M + H]^+^ = 344 at 5.9 min is shown in Figure S3 and it is compatible with a further oxidized product. The addition of 14 amu (atomic mass unit) to the parent can be attributed to the formation of a keto group. 

The parent [M + H]^+^ = 330 and the metabolites [M + H]^+^ = 346 and [M + H]^+^ = 344 were also detected in the mouse plasma samples collected after oral treatment of CD1 mice with 50 mg/kg of (−)-AM1 (Figure S4).

### 2.5. P450 and hERG Interaction

The potential for inhibition of the major human P450 isoenzymes was also investigated. Racemic AM1 and the two enantiomers all showed a similar profile. At 3 µM, all compounds caused strong inhibition (>60%) of the CYP2D6 activity, with only a slight inhibition of the remaining four isoform activities (Table 4). It is worth mentioning that CYP2D6 is the only P450 isoenzyme which is also inhibited by CQ (Table 4) and confirmed in drug–drug interaction studies in humans [28].

The 4-aminoquinoline class of molecules is associated with potential cardiovascular side effects. Preliminary experiments of hERG binding indicated that (±)-AM1 and (−)-AM1 inhibit hERG with a Ki in the range of 0.5–0.8 µM, whilst chloroquine in the same experiments had a Ki of 2.2 µM. Electrophysiological functional assays will be required to further exclude this liability.

### 2.6. Pharmacokinetics

Table 5 reports the kinetic parameters evaluated after oral administration 10 mg/kg of the AM1 racemate and its enantiomers to CD1 mice. No significant differences in maximal plasma concentration achieved among the racemate or its enantiomers were observed. Cmax was about 0.10 μM for all compounds and the AUC 20 min·µM after oral administration of 10 mg/kg. 

The (−)-AM1 enantiomer was also orally injected to the mice at three increasing doses (10, 25, and 50 mg/kg) and dose proportionality was displayed, together with a parallel kinetic of elimination of the three treatments, independently from the given dose (Figure S5).

## 3. Discussion

Historically, the 4-aminoquinolines are a very successful chemical class of antimalarials, which includes chloroquine and amodiaquine, still clinically used against *P. vivax* infection or in combination therapy against *P. falciparum*. In addition to their direct antiparasitic activity, the advantages of most of the 4-aminoquinolines are stability, high bioavailability, favorable PK/PD properties after oral administration, and low cost. For these reasons, although the emergence and spread of resistant *P. falciparum* strains significantly affected the use of chloroquine as monotherapy, research on a new generation of 4-aminoquinolines effective against multiresistant *P. falciparum* strains never stopped and novel compounds are presently in development.

This is the case for ferroquine and for AQ-13, in which a ferrocenyl moiety or a short diethylaminopropyl group replaced the side chain of chloroquine, respectively [16,29,30]. Both compounds successfully completed phase I studies [19,20] in human volunteers and in asymptomatic patients, and are presently under phase II efficacy studies, either alone or in combination (https://clinicaltrials.gov/ct2/show/NCT01614964, https://clinicaltrials.gov/ct2/show/NCT02497612).

In both cases, the ability to overcome CQ resistance has been attributed to the modification of the lateral side chain of the 4-aminoquinoline ring, with the introduction of the ferrocene moiety as in ferroquine, or by reducing its length as in AQ13. Similarly, the compound AM1, described in this paper, was obtained by the introduction of a bulky, basic, and lipophilic quinolizidine ring (*N*-lupinyl) in the lateral chain of a 7-chloro-4-aminoquinoline [22], leading to a compound more lipophilic than CQ (AM1 LogD_7.4_ = 2.26 vs. CQ LogD_7.4_ = 0.90) [23]. Differently from the semisynthetic product which used (−)-lupinine as starting material, the total synthesis of AM1 lead to the formation of a racemate, which was then tested for antimalarial activity and toxicity in vitro together with the isolated enantiomers. The total synthesis of (±)-AM1 is cheap and straightforward, and can be accomplished in four steps using commercially available material [25]. 

The AM1 enantiomers displayed in vitro activity similar to the racemate, which supports further studies on the in vivo efficacy of these compounds and on the analysis of their pharmacokinetic and ADME/tox properties.

Here, we report the activity of (±)-AM1 and its enantiomers in the low nanomolar range documented against laboratory chloroquine-resistant *P. falciparum* strains, as well as the multidrug resistant strain TM91C235 strain from Thailand. In combination studies with DHA, (−)-AM1 displayed an additive effect similar to that obtained with CQ and DHA in combination. Therefore, the profile of the compound appears good in terms of possible administration against CQ-resistant strains and in combination with artemisinin.

Moreover, (±)-AM1 displayed an inhibitory activity three-fold higher than chloroquine against ten field isolates of *P. vivax*, an infection that accounts for approximately 4% of malaria cases worldwide, 41% of which occur outside Africa [1]. The WHO’s recommendation for the treatment of *P. vivax* infections is chloroquine, in non-chloroquine resistant areas, or ACT. However, in areas where chloroquine-resistant *P. vivax* infections have been identified, ACT should be the preferred choice. All ACTs, except the artesunate + sulfadoxine-pyrimethamine (AS + SP) combination, are demonstrated to be effective against the blood-stage infections of *P. vivax*. The in vitro and in vivo data reported for (±)-AM1 support its possible development against *P. vivax,* as well.

The AM1 racemate and its enantiomers were orally active at suitable doses in the classical in vivo four-day test in mice as schizonticidal agents, with no significant differences in potency among (±)-AM1 and its enantiomers. They were inactive against the parasite transmission stages in the *P. berghei/A. stephensi* model. These results are consistent with previous data on this class of drugs [31]. However, for other 4-aminoquinolines recently synthesized (TDR58445 and TDR58446), a reduction of activity in both early and late gametocytes stages has been described [32], although at a relatively higher concentration (1 µM), compared to the activity on asexual stages. 

In terms of in vitro metabolic stability and interference with the P450 enzymes, racemic AM1 and its enantiomers did not differ significantly from chloroquine, used as reference drug. Similarly, the binding assay to hERG indicated that these molecules may share a potential liability for cardiovascular side effects with the quinoline antimalarials, including chloroquine and piperaquine, currently recommended by WHO for malaria therapy [33]. In the case of future developments, more stringent methods of analysis will be necessary.

PK studies in animals were performed by oral gavage according to the target product profile for an antimalarial drug. The need for a complex formulation (SSV) was dictated by the poor solubility of the free bases, which could also have an impact on total drug disposition.

This information is of relevance in respect to the in vivo efficacy data. In fact, all efficacy experiments have been performed with the compounds prepared as free bases, suggesting that the in vivo data obtained so far could underestimate the potential of the test compound as antimalarial.

The introduction of a bulky bicyclic tertiary amine in place of the diethylaminoalkyl chain typical of CQ and AQ13 changed the metabolic profile, thus *N*-dealkylation is not the preferred metabolic pathway. This aspect could be important because it has been demonstrated that the major CQ metabolites, desethylchloroquine (DEC) and, overall, bisdesethylchloroquine (bisDEC), are less active than the parent compound on CQ-resistant strains of *P. falciparum* [34]. Similarly to CQ, in vivo studies on AQ13 metabolites showed that its *N*-deethylation led to specific changes in lipophilicity and increased the cross-resistance of this compound even more than that of CQ [35]. The main AM1 metabolite seems to be an *N*-oxide on the amino methylene bridge. In terms of metabolic stability, this difference does not increase the half-life of AM1 compared to CQ, but could change the in vitro antimalarial profile. It remains to be elucidated how the in vivo antimalarial activity of AM1 is influenced by these metabolites.

In conclusion, we have demonstrated that the 4-aminoquinoline compounds remain of high interest as antimalarial agents, and AM1 in particular displays a very attractive profile in terms of in vitro and in vivo activity.

## 4. Materials and Methods

### 4.1. Compounds

Racemic AM1 and its enantiomers (Figure 1) were synthesized as previously described [25,36]. Chloroquine and all the other reagents, unless indicated, were purchased from Sigma (Sigma Italia, Milan, Italy).

### 4.2. Cultures of P. falciparum and In Vitro Chemosensitivity Assays 

The strains D10 and 3D7 (CQ-Sensitive), and W-2, FCR1, FCR3 (CQ-Resistant), and TM91C235 (multidrug-resistant) of *P. falciparum* were cultured in vitro as described by Trager and Jensen with minor modifications [37]. Briefly, parasites were maintained at 5% hematocrit (human type A+ red blood cells) in RPMI 1640 (EuroClone, Celbio, Pero, Italy) medium with the addition of 10% heat-inactivated human serum, 20 mM Hepes, and 2 mM glutamine (EuroClone). The cultures were maintained at 37 °C in a standard gas mixture consisting of 1–3% O_2_, 5% CO_2_, and 92–94% N_2_. Compounds were dissolved in either water or DMSO and then diluted with medium to achieve the required concentrations (final DMSO concentration <1%, which is non-toxic to the parasite). Asynchronous cultures of *P. falciparum* with parasitaemia of 1–1.5% and 1% final hematocrit were aliquoted into 96-well flat-bottom microplates (COSTAR) with serial dilutions of test compounds, and incubated for 72 h at 37 °C. Parasite growth was determined spectrophotometrically (OD650) by measuring the activity of the parasite lactate dehydrogenase (pLDH) according to a modified version of the method of Makler, in control and drug-treated cultures [38]. The antimalarial activity is expressed as 50% inhibitory concentrations (IC_50_); each IC_50_ value is the mean and standard deviation of at least three separate experiments performed in duplicate [22].

The antimalarial activity of (−)-AM1 was assayed in combination with dihydroartemisinin (DHA) against W-2 (CQ-R) or D10 (CQ-S) strains of *P. falciparum* by potentiation tests as previously described [39]. CQ was used as a control. Isobolograms were constructed by plotting a pair of fractional IC_50_ values for each combination of (−)-AM1 or CQ with DHA [40]. 

An isobologram close to the diagonal indicates an additive effect. Curves significantly (>2) above or below the diagonal indicate antagonistic or synergistic effects, respectively. 

### 4.3. In Vitro Assays against P. vivax Field Isolates

Ten *P. vivax* isolates were collected from June to July 2010 from malaria patients attending the Shoklo Malaria Research Unit (SMRU) clinics, Mae Sot region of the Tak Province in north-western Thailand under the ethical guidelines in the approved protocol OXTREC 027-05 (Centre for Clinical Vaccinology and Tropical Medicine, University of Oxford, Oxford, UK). These samples were collected from patients with no prior antimalarial therapy and with microscopically confirmed *P. vivax*. After written consent, blood samples were collected by venepuncture in 5 mL volume lithium heparinized tubes, which were then transported to the laboratory at SMRU within 5 h of collection. The ten isolates were chosen for testing because the majority (>80%) of the parasites were at the mid trophozoite stage (around 20 h post invasion) and no schizonts were detected. After platelet and leukocyte removal [41], the susceptibility of these *P. vivax* isolates to AM1, chloroquine diphosphate (MW 515.9, Sigma-Aldrich^®^, Milan, Italy), and artesunate (Base MW282.3, Holly Pharmaceuticals Co Ltd., Tustin, CA, USA) was assessed as previously described [42,43].

### 4.4. In Vivo Efficacy Tests and Transmission-Blocking/Prophylactic Activities

In vivo antimalarial efficacy tests were performed using *P. berghei* (CQ sensitive, CQ-S) ANKA and *P. yoelii* (CQ resistant, CQ-R) rodent species in the standard four-day suppressive Peters’ test [26]. Groups of five CD1 mice were inoculated i.v. with 4 × 10^6^ infected erythrocytes/mice. The compound, dissolved in standard suspending formula (SSV) (0.5% sodium carboxymethylcellulose, 0.5% benzyl alcohol, 0.4% Tween 80, 0.9% NaCl), was administered orally, once a day for a period of four days, starting two hours post-infection. Four drug doses were used, ranging from 1 to 30 mg/kg to determine ED_50_ and ED_90_ values. Parasitaemia was determined by microscopic examination of Giemsa-stained blood films taken on day four, processed using MICROSOFT^®^ EXCEL spreadsheets (Microsoft Corp., Redmond, WA, USA), and expressed as percentages of inhibition from the arithmetic mean parasitaemias of each group in relation to the untreated group. ED_50_ and ED_90_ values were calculated by GraphPad Prism 6 (GraphPad Software Inc, La Jolla, CA, USA).

(−)-AM1 was also tested in transmission-blocking and prophylactic studies using the *P. berghei*/*A. stephensi* model as described, with a few modifications [44].

For the transmission-blocking test, four BALB/c female mice (18–20 g) were infected i.p. with 10^7^ *P. berghei*-infected red blood cells. Four days later, once confirmed for gametocyte carriage, two mice were treated with 50 mg/kg (−)-AM1, through the oral administration of 500 µL of a methylcellulose suspension, obtained by dissolving a 20 mg/mL ethanol stock solution in 0.5% methylcellulose to a final 9% ethanol concentration. Two mice received the vehicle only as controls. One hour after the treatment, mice were anesthetized and exposed to the bites of 100 sugar-starved female mosquitoes, at 20 °C. Ten days after the blood meal, samples of mosquito midguts from each treatment (*n* = 40) were dissected and *P. berghei* oocysts were manually counted using light microscopy at 400× magnification. Oocyst numbers were log-transformed and the geometric means were compared using the Student’s *t*-test.

For the prophylactic test, the experimental drugs were administered at 50 mg/kg per os for 4 consecutive days; 90 min after the last administration, mice were infected by 6–10 infective mosquito bites. Thick and thin Giemsa-stained smears were prepared and examined on days 5–10 post-infection to assess parasite density per μL of blood.

Rearing and handling of experimental animals was in full compliance with the Italian Legislative Decree on the “protection of animals used for experimental and other scientific purposes” (D. Lgs. 26 of 03/04/2014) and in full adherence with the European Directive 2010/63/UE.

### 4.5. LC–MS/MS Analytical Methods

Samples from the metabolic stability and metabolism studies were analyzed under the following conditions: UPLC interfaced with a Premiere XE triple Quadrupole Waters for stability studies, and Bruker Daltonics Ion Trap HCT Ultra for metabolic studies. A gradient from 2% to 100% (Water, MeOH, 5 mM Ammonium Acetate) over 2 min (stability) or 12 min (metabolism) in a C18 BEH (dimensions 1 by 50 mm for stability and 2.1 by 100 mm for metabolism, 1.7 µm column, 45 °C temperature, flow rate 0.5 mL/min) was used. For stability studies, the detection was performed in MRM (ESI positive, Q1/Q3 330.16/152.00, Cone Voltage 45 V; Extractor 5 V; Capillary 3.4 kV), while for metabolism studies, analysis was performed in Auto MS/MS mode (ESI positive; Capillary 109 V; Dry Temp 350 °C; Nebulizer 30 psi; Dry gas 10 L/min; HV capillary 500 V; Mass Accu time 500 µs; Mass range MS scan 100–600 amu, MS/MS 50–450 amu).

Samples from the pharmacokinetic studies were analyzed under the following conditions: UPLC interfaced with a Premiere XE triple Quadrupole, Waters. A gradient from 2 to 100% (Water, MeOH, 5 mM Ammonium Acetate) over 1.3 min was used in Acquity HSS T3 (dimensions 2.1 by 50 mm, 1.8 µm column, 40 °C temperature, flow rate 0.5 mL/min). Detection was performed in MRM (ESI positive, Q1/Q3 330.16/152.00, Cone Voltage 45 V; Extractor 5 V; Capillary 3.4 kV). The limit of detection was 1 ng/mL. Pharmacokinetic parameters were determined by Non-Compartmental Analysis, using the linear trapezoidal rule and uniform weight.

### 4.6. In Vitro Microsome Stability and Metabolism Studies

For stability and metabolism studies, test compounds were dissolved in DMSO, diluted at the final concentration of 1 µM, and pre-incubated in duplicate for 10 min at 37 °C in potassium phosphate buffer, pH 7.4, 3 mM MgCl_2_, with rat, mouse, and human hepatic microsomes (Xenotech Europe, Pfungstadt, Germany) at the final concentration of 0.5 mg/mL. The reaction was then started by adding the cofactor mixture of NADP, Glc6P, and G6P-DH, in the presence of UDPGA 1 mM in human microsomes to promote glucuronide formation. Acetonitrile (with verapamil as internal standard) was added to the samples at the times time 0 and 30 min to stop the reaction. Samples were then centrifuged and the supernatants analysed and quantified by LC-MS/MS. Reference compounds of known metabolic stability, 7-ethoxycoumarin (7-EC) and propranolol, were used as controls.

The percentage of the area of test compounds remaining after 30 min incubation was calculated in respect to the area of compounds at time = 0 min. A compound showing a % remaining greater than 95% at 30 min was considered stable. In biotransformation studies after, the full scan acquisition data were processed by MetaboLynx software (Waters Corp., Milford, MA, USA) comparing the signals at times 0 and 30 min. Samples were re-analyzed by acquiring the daughter scan of the potential metabolites found during the full scan acquisition.

### 4.7. Pharmacokinetics

Pharmacokinetic data were collected in mice by testing the racemate and the enantiomers at the same single dose, and vehicle-utilized for in vivo studies (see composition in paragraph 4.4). Briefly, male CD-1 mice (Charles River Laboratories) weighing 18–22 g received single dose (10 mg/kg) oral administrations of the AM1 racemate and single enantiomers, each dissolved in SSV. For each experiment, twenty-four mice/dose and three mice/time-point were used. Blood samples were collected at eight selected time-points (15, 30 and 60 min; 2, 4, 6, 8 and 24 h) under ethyl ether anaesthesia. The heparinized tubes containing blood samples were immediately placed on ice and centrifuged, and the supernatant collected and frozen at −80 °C prior to analysis. Pharmacokinetic plasma samples (spiked with internal standard) were filtered (Sirocco filter plate, Waters) under vacuum, and 5 µL analysed by LC-MS/MS.

### 4.8. P450 Assay

Potential inhibition of the five most important human P450 isoforms (CYP1A2, CYP2C9, CYP2C19, CYP2D6, and CYP3A4) was measured using BD Gentest assays using specific substrates that become fluorescent upon CYP metabolism [45]. Compounds, dissolved in DMSO, were tested in duplicate (*n* = 2) at 3 μM in a 96-well plate, as described [46]. Specific selective isoform inhibitors were added in each experiment as controls. The data were expressed as percentage inhibition in respect to the control without inhibition (<5% inhibition was considered no effect), and for standards, the IC_50_ (concentration causing 50% inhibition) was then determined by using Grafit v. 5.0.1 (Erithacus Software Ltd., Horley, Surrey, UK).

### 4.9. hERG Binding Assay

[^3^H]-astemizole (PerkinElmer ITALIA Spa, Milan, Italy) radioligand binding assays were performed in HEK293 cells membranes stably transfected with the hERG (U04270); non-specific binding was evaluated in the presence of an excess of 10 µM cold astemizole. Experiments were performed at 22 °C for 60 min in triplicate and compounds tested in a range from 0.03 µM to 30 µM. Two separate experiments were performed and data analyzed with non-linear fitting using a four-parameter logistic [47].

## Figures and Tables

**Table 1:** In vitro antimalarial activities of the AM1 racemate and its enantiomers on *P. falciparum* laboratory strains and *P. vivax* field isolates.

Compounds	*P. falciparum* IC_50_ (nM) ^a^					*P. vivax* IC_50_ (nM) ^b^
	D10 ^c^ (CQ-S)	W-2 ^c^ (CQ-R)	TM91C235	FCR1	FCR3	Field Isolates
(±)-AM1	16.15	35.44	-	-	-	29.72
(−)-AM1	23.98	53.67	16.74	32.87	23.28	-
(+)-AM1	17.49	35.77	-	-	-	-
CQ	23.72	437.62	199.46	181.93	112.56	86.66

^a^ The assay was evaluated after 72 h incubation, using the parasite lactate dehydrogenase (pLDH) method, mean of 3 different experiments. ^b^ IC_50_ results from 10 field isolates from the Thai–Burmese Border. ^c^ Data already published in [25].

**Table 2:** Therapeutic efficacy of (±)-AM1, (−)-AM1, (+)-AM1, and chloroquine against *P. berghei* ANKA and *P. yoelii* infection in mice using the Standard four-day Test.

Compounds	*P. berghei* ^a^		*P. yoelii* ^a^	
	ED_50_ (95% CI) (mg/kg)	ED_90_ (95% CI) (mg/kg)	ED_50_ (95% CI) (mg/kg)	ED_90_ (95% CI) (mg/kg)
(±)-AM1	2.77 (1.26–4.29)	12.58 (7.69–17.46)		
(−)-AM1	2.05 (1.31–2.79)	8.65 (7.06–10.23)	1.55 (1.25–1.92)	4.95 (3.08–7.94)
(+)-AM1	1.60 (1.08–2.11)	2.03 (1.46–2.60)		
Chloroquine	1.12 (0.98–1.26)	2.93 (2.64–3.22)	<3	58.5 (5.38–6.42)

^a^ CD1 mice were inoculated i.p. with 4 × 10^6^ infected erythrocytes/mouse. Four drug doses of compounds, dissolved in standard suspending formula (SSV), were administered orally, once a day for four days, starting two hours post-infection. Percent parasitaemia was evaluated by microscopic examination of Giemsa-stained smears taken on day 4. ED_50_ and ED_90_ values were calculated by GraphPad Prism6. All animal work at The London School of Hygiene and Tropical Medicine (LSHTM) was conducted under licence (PPL70/8427) according to UK Home Office regulations: The Animals (Scientific Procedures) Act of 1986.

**Table 3:** In vivo and in vitro main metabolites of (−)-AM1 and their proposed structures.

MH^+^ (rt, min)	Proposed Structure	Metabolic Products Detected In Vitro and In Vivo			
		In Vitro Microsomes (−)-AM1 1 μM			In Vivo Plasma Levels after 50 mg/kg Oral Gavage
		Human	Rat	Mouse	Mouse
		At 30 Min			At 120 Min
330 (6.1)	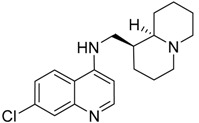	+	+	+	+
344 (5.9)	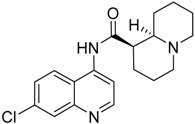	+	+	+	ND
346 (4.7)	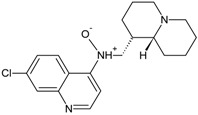	+	+	+	+

rt: retention time; ND: Not detectable.

**Table 4:** Comparative P450 inhibition potential of racemic AM1, its enantiomers, and chloroquine.

Compounds	P450 Interaction—Gentest Kit				
	CYP1A2	CYP2C9	CYP2C19	CYP2D6	CYP3A4
	CEC ^a^	MFC	CEC	AMMC	BFC
	Mean % Inhibition at 3 µM ^b^				
(±)-AM1	<5	5.7 ± 0.2	6.0 ± 1.6	61.4 ± 2.0	11.6 ± 0.4
(+)-AM1	8.3 ± 0.8	11.1 ± 0.5	9.0 ± 1.1	62.2 ± 1.0	14.3 ± 1.9
(−)-AM1	<5	<5	5.0 ± 1.3	74.4 ± 3.5	8.4 ± 0.8
CQ	<5	<5	<5	20.4 ± 1.1	6.8 ± 1.7

^a^ Substrates: CEC: 3-Cyano-7-ethoxycoumarin; MFC: 7-Methoxy-4-trifluoromethylcoumarin; AMMC: 3-[2(*N*,*N*diethyl-*N*-methylamino)ethyl]-7-methoxy-4-methylcoumarin; BFC: 7-Benzyloxy-4-(trifluoromethyl)-coumarin. ^b^ Results are expressed as Mean ± S.D., *n* = 2; <5% inhibition is considered no effect.

**Table 5:** Pharmacokinetic parameters of the AM1 racemate and its enantiomers.

Parameters ^a^	(±)-AM1	(+)-AM1	(−)-AM1
	10 mg/kg		
Cmax (µM)	0.0996	0.108	0.084
Cmax (ng/mL)	32.78	35.48	27.60
Tmax (min)	30	120	15
MRT last (min)	196	162	200
AUC last (min·µM)	19.5	22.9	18.8
AUC last (min·ng/mL)	6421	7551	6179

^a^ Pharmacokinetic parameters were determined by the non-compartmental analysis WinNonlin 5.1, linear trapezoidal, uniform weight. The following parameters were calculated: Cmax (maximum plasma concentration), Tmax (time take to reach Cmax), MRT (mean residence time), AUC (area under the curve). In vivo PK experiments were performed with male CD-1 mice orally-treated by a single dose (10 mg/kg) of the AM1 racemate and single enantiomers, each dissolved in SSV. Blood samples were collected at eight selected time-points (15, 30, and 60 min; 2, 4, 6, 8, and 24 h) under ethyl ether anaesthesia, and plasma samples analyzed by LC/MS/MS.

## Supplementary Materials

The following are available online.
