# Supplementary material for: In Vivo and In Vitro Activities and ADME-Tox Profile of a Quinolizidine-Modified 4-Aminoquinoline: A Potent Anti-P. falciparum and Anti-P. vivax Blood-Stage Antimalarial
Source: Molecules. 2017 Dec 1;22(12):2102. doi: 10.3390/molecules22122102 (PMC6149971; doi:10.3390/molecules22122102)
Supplement: Supplementary file 1 [file molecules-22-02102-s001.pdf]

Figure S1 (Supplementary information). Metabolic profile of (-)-AM1 with mouse microsomes after 30 min incubation

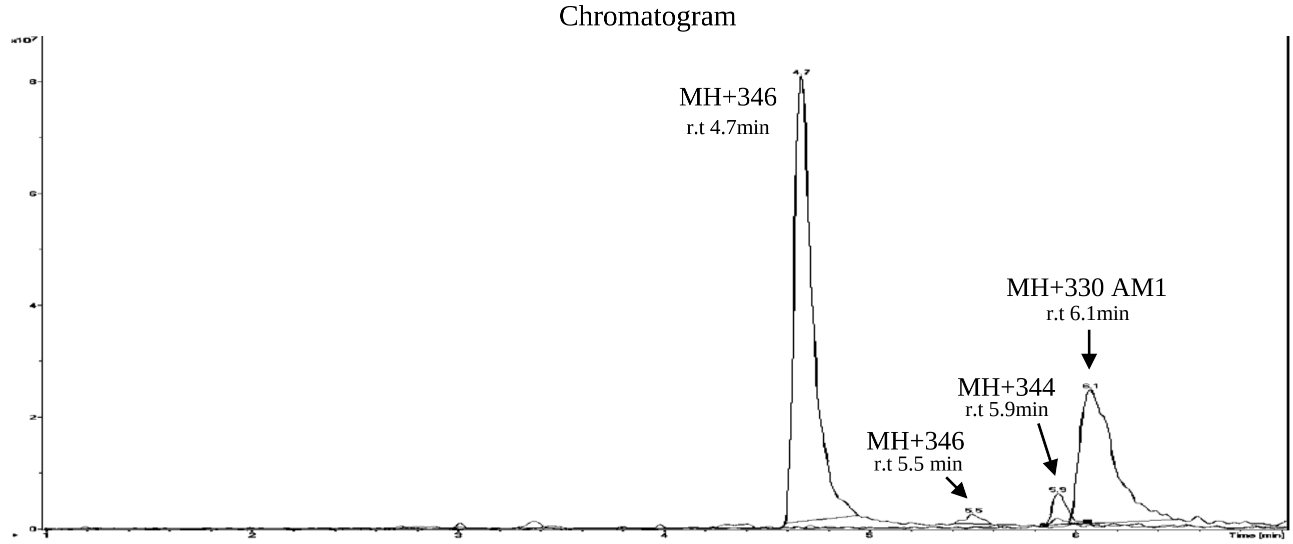

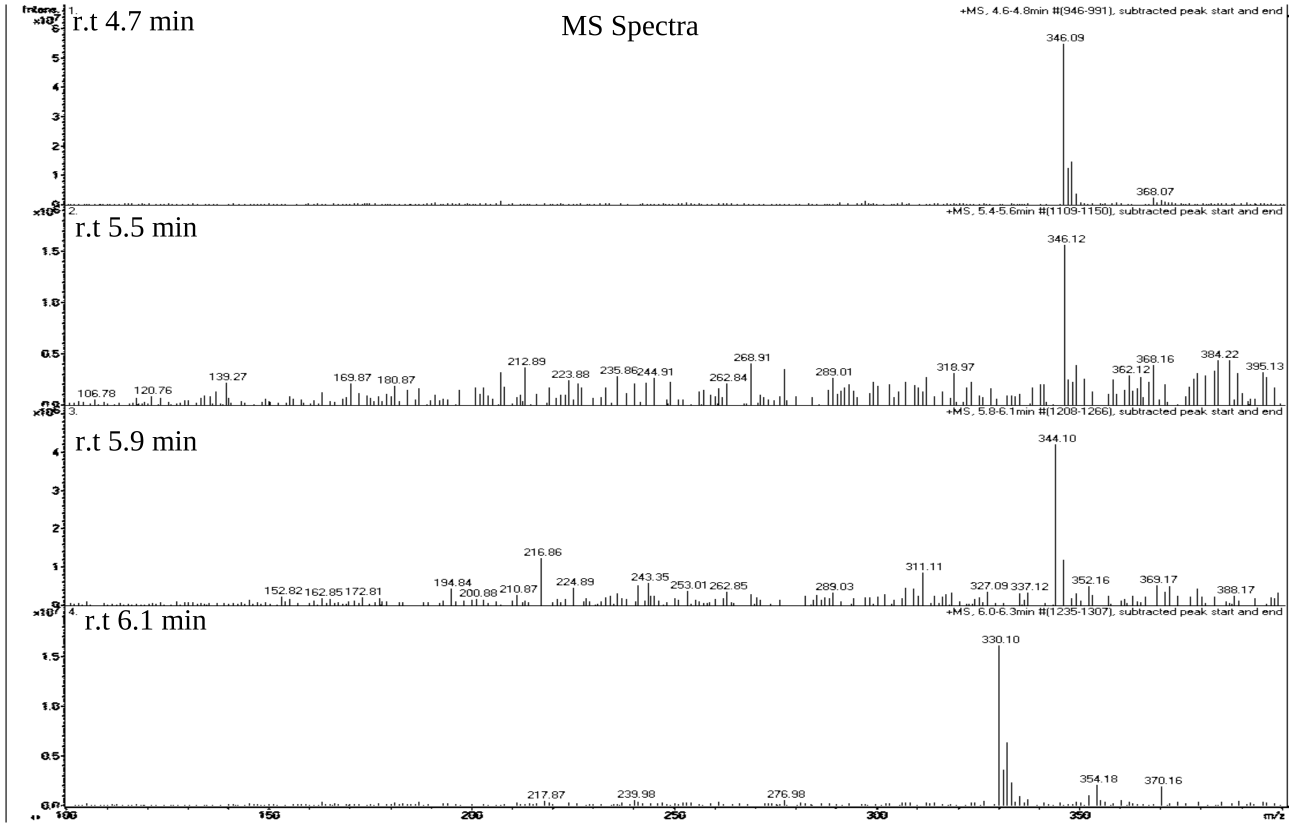

Figure S2 (Supplementary information). MS/MS spectra of the metabolite at 4.7 min MH+346 and attribution of the major fragments

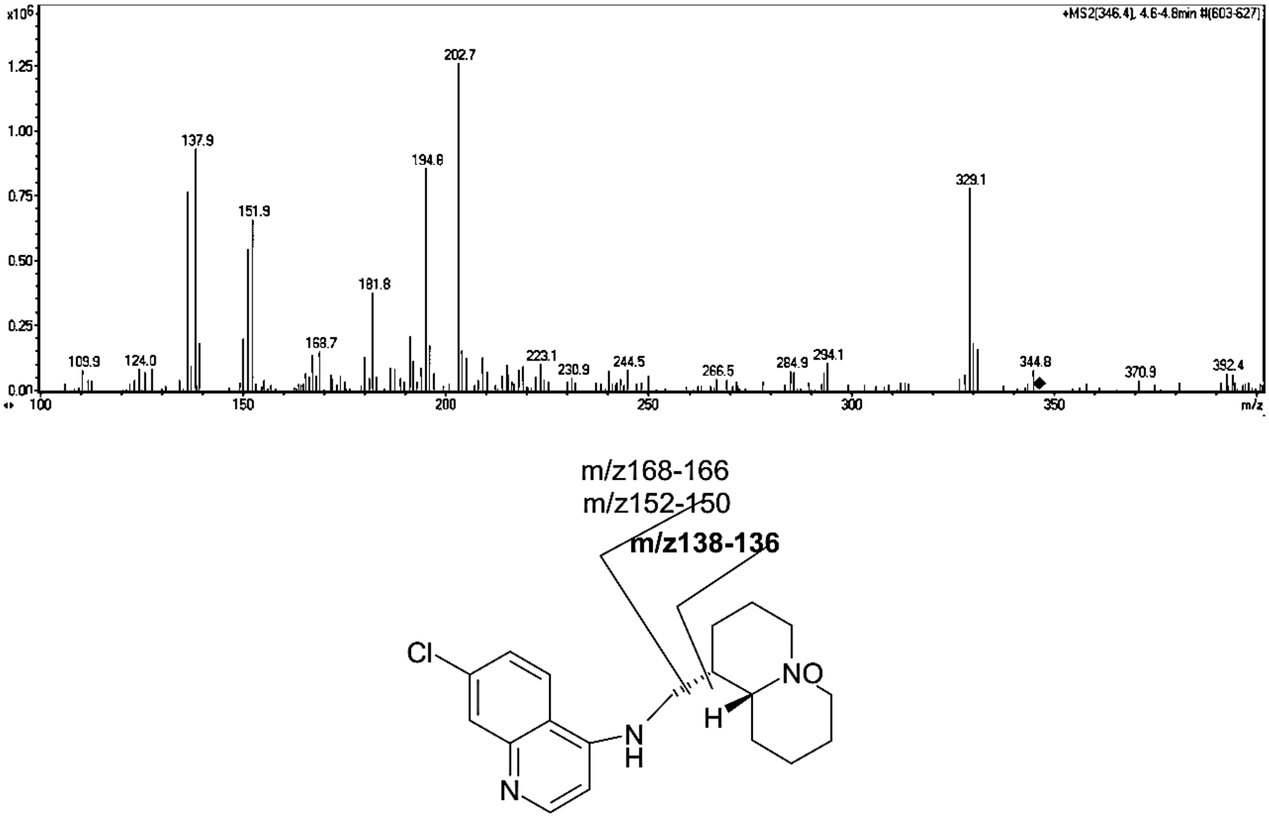

Figure S3 (Supplementary information). MS/MS spectra of the metabolite at 5.9min MH+344 and attribution of the major fragments

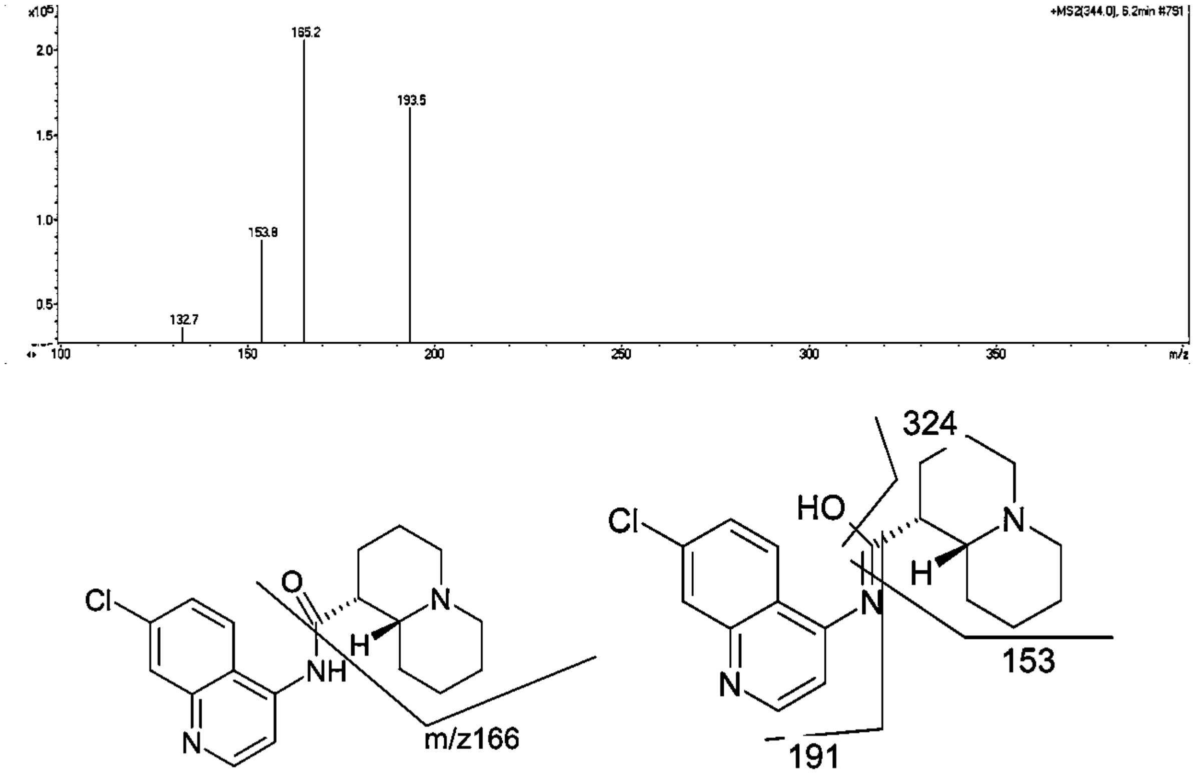

Figure S4 (Supplementary information). Metabolite profile of AM-1 in the mouse plasma after 120 min from oral administration of 50 mg/kg

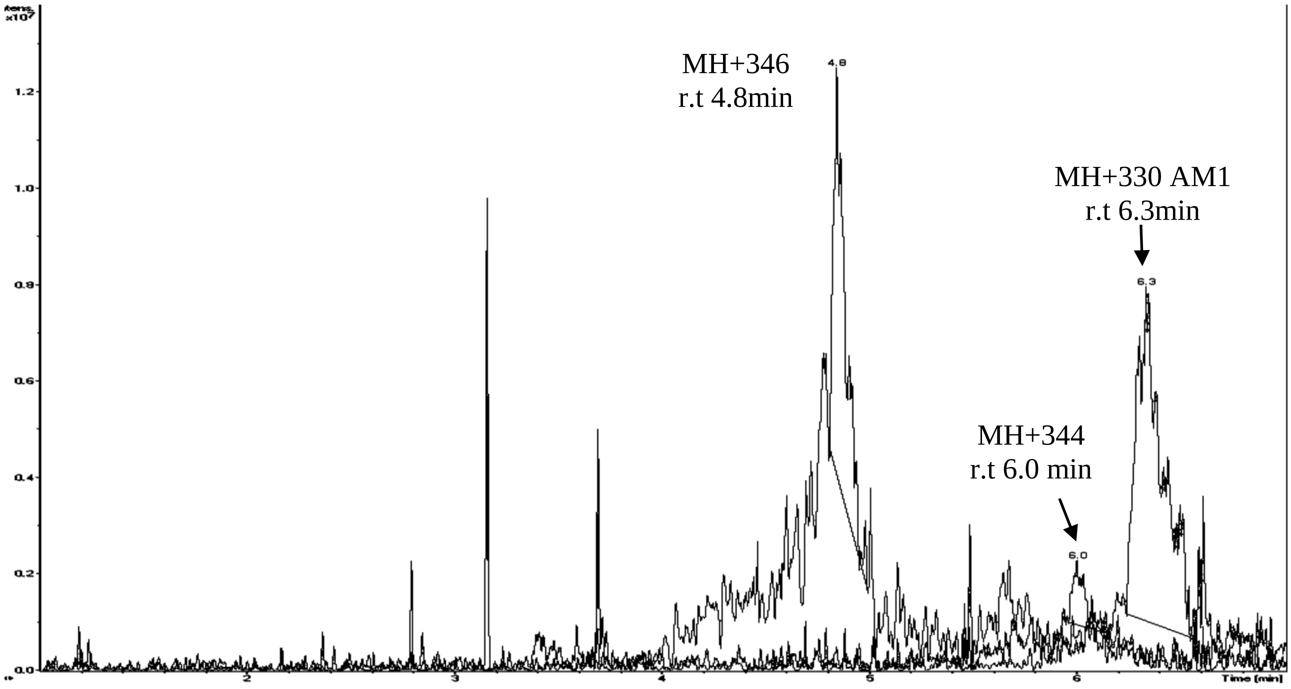

Figure S5. Pharmacokinetics of (-)-AM1 in mice: dose proportionality

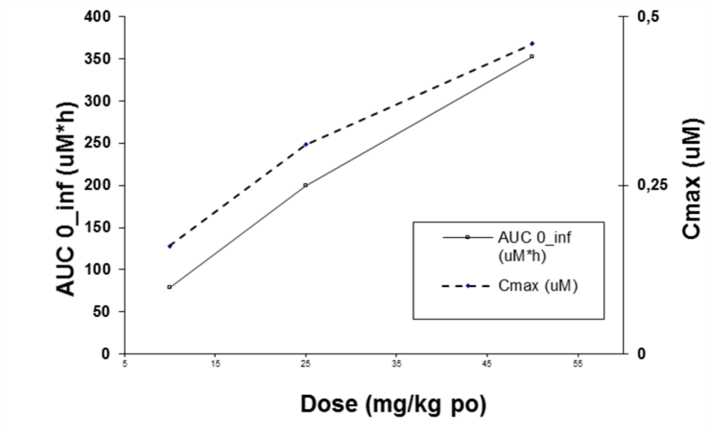

In vivo PK experiments were performed with male CD-1 mice orally-treated by three doses (10, 25 and 50 mg/kg) of (-)-AM1, dissolved in SSV. Blood samples were collected at eight selected time-points (15, 30 and 60 min, 2, 4, 6, 8 and 24 h) under ethyl ether anaesthesia, and plasma samples analyzed by LC/MS/MS.
